# Recurrence of hepatitis C virus during leucocytopenia and spontaneous clearance after recovery from cytopenia: a case report

**DOI:** 10.1186/1752-1947-1-169

**Published:** 2007-12-04

**Authors:** Norbert H Gruener, Bijan Raziorrouh, Maria-Christina Jung

**Affiliations:** 1 Medical Department II, Klinikum Großhadern Ludwig-Maximilians-University, Munich, Germany

## Abstract

**Introduction:**

There is little information about the risk of HCV recurrence in immunosuppressed patients. Although the presence of antibodies to HCV and the absence of HCV-RNA is usually considered to indicate viral elimination, the virus may not be completely eliminated but may be under control of an effective immune response.

**Case presentation:**

A 69 year old man presented with jaundice, elevated ALT, AST, lipase and concomitant abdominal pain. He was found to be positive for HCV-RNA (genotype 3a) and was diagnosed with acute hepatitis C. Six weeks later no HCV-RNA was detected, and the patient was diagnosed with hyperthyreosis and started on propylthiouracil. After 4 weeks of propylthiouracil treatment, the patient developed leucocytopenia, followed by liver function deterioration and reappearance of HCV-RNA. Propylthiouracil was discontinued and his leukocyte counts increased. Twenty-eight weeks after onset of acute hepatitis C, no HCV-RNA was detected.

**Conclusion:**

This case history shows the risk of recurrence of HCV during leucocytopenia. These findings indicate that patients who are anti-HCV positive but HCV-RNA negative may be at risk of cytopenia-induced HCV reactivation.

## Introduction

Spontaneous clearance of hepatitis C virus is highly associated with a strong T lymphocyte response [1,2]. It is unclear, however, if the virus is completely eliminated or is present but under the control of the immune response. Analogous to other viral diseases (e.g. HBV), it is likely that traces of the virus remain in the body [3-5]. We describe here the case of a 69 year old man with acute hepatitis C virus infection who developed propylthiouracil-induced leucocytopenia, followed by cytopenia-induced HCV recurrence. With recovery of leucocytes the virus was again cleared spontaneously. These findings suggest that agranulocytosis can lead to reactivation of hepatitis C virus and that recovery from hepatitis C is not equivalent to complete viral clearance. Therefore HCV reactivation has to be considered in the diagnosis of patients positive for anti-HCV antibodies but negative for HCV-RNA who experience leucocytopenia for any reason.

## Case presentation

A 69 year old man was admitted to an external hospital due to jaundice. On the basis of cholestatic laboratory findings with elevated lipase and concomitant abdominal pain, an endoscopic retrograde cholangiopancreatography (ERCP) was performed. Trapped air in CT scan indicated a small perforation after papillotomy. In addition the patient developed post ERCP pancreatitis with signs of peritonitis. He was therefore admitted to our intensive care unit. At admission the following laboratory parameters were out of range: ALT 1637 U/l (normal range < 45 U/l), AST 717 U/l (normal range < 40 U/l), and bilirubin 19 mg/dl (normal range < 1 mg/dl). During the following days, his prothrombin time dropped to 34%. He was subsequently shown to be positive for HCV-RNA (genotype 3a) and diagnosed with acute hepatitis C. Other coexisting infections, metabolic or toxic liver diseases were ruled. The patient recovered from the perforation and pancreatitis without surgical intervention.

Six weeks after onset of acute hepatitis, he was negative for serum HCV RNA (Table 1). At this time, he was diagnosed with hyperthyreosis and started on propylthiouracil 50 mg three times daily. The patient developed leucocytopenia, followed by worsening of liver function, 10 weeks after the onset of acute hepatitis (Table 1). His AST and ALT were normal, but his bilirubin had increased. Propylthiouracil treatment was stopped and his leukocyte count recovered at week 12. Twenty weeks after onset of acute disease, HCV-RNA was detected, at a concentration of 140,000 IU/ml, at which time his AST (388 U/ml) and ALT (371 U/ml) were increased markedly. Seven weeks later, he was again negative for HCV-RNA without antiviral treatment, and his liver enzymes had again normalized. 47 weeks after onset of acute hepatitis HCV-RNA was still negative indicating sustained spontaneous viral clearance.

**Table 1:** Selected blood test results. Values at the beginning of acute hepatitis C and during the 47 week follow-up period (n.t. = not tested)

Laboratory value (normal range)	Week 1	Week 6	Week 10	Week 21	Week 28	Week 47
Leukocytes (4.0–11.0 G/l)	19.8 G/l	9.0 G/l	0.6 G/l	4.7 G/l	5.3 G/l	5.1 G/l
ALT (< 45 U/l)	1637 U/l	119 U/l	35 U/l	371 U/l	25 U/l	27 U/l
AST (<40 U/l)	717 U/l	103 U/l	23 U/l	388 U/l	25 U/l	21 U/l
Bilirubin (<1.0 mg/dl)	19.0 mg/dl	11.4 mg/dl	7.9 mg/dl	1.8 mg/dl	1.1 mg/dl	0.9 mg/dl
Prothrombin time (70–100%)	44%	65%	50%	65%	70%	75%
HCV-RNA (negative)	2.300.000 IU/ml	negative	n.t.	140.000 IU/ml	negative	negative

Retrospective our diagnosis of acute hepatitis C was therefore supported by several data. Patients wife was diagnosed with acute hepatitis C two weeks later. During follow-up both had a documented spontaneous viral elimination. As a possible mode of infection both had made an ozone therapy at an alternative practitioner several weeks before onset of acute disease.

## Discussion and conclusion

Anti-HCV positive patients without HCV-RNA (>6 months) are considered cured of hepatitis C. These patients have either cleared the virus spontaneously or after treatment. It is generally believed that hepatitis C virus is not completely eliminated from the body but is under control of the immune system. HCV-RNA has been reported to be present in the liver and peripheral blood mononuclear cells [3-5] of patients with no detectable serum HCV-RNA. The clinical relevance of this phenomenon is unclear. Chemotherapy has been shown to increase serum transaminase concentrations in HCV-RNA positive patients, an increase thought to be due to immunosuppression [6]. To date, however, only one case report has described HCV RNA recurrence after chemotherapy [7]. Our case shows the risk of HCV recurrence due to leucocytopenia caused by reasons other than chemotherapy. Patients positive for anti-HCV antibodies but negative for HCV-RNA are therefore at risk of HCV reactivation due to cytopenia. Our findings also emphasize the importance of virus specific T cell responses in viral diseases. Thus, HCV recurrence should be considered in the differential diagnosis of anti-HCV positive patients who experience cytopenia.

In summary, our findings indicate that greater attention should be paid to liver function tests, not only in patients chronically infected with HCV but in HCV-RNA negative patients with a previous history of hepatitis C virus infection.

## List of abbreviations

HCV: Hepatitis C virus:

RNA: Ribonucleic acid:

ALT: Alanine aminotransferase:

AST: Aspartate aminotransferase:

ERCP: Endoscopic retrograde cholangiopancreatography.

## Competing interests

The author(s) declare that they have no competing interests.

## Authors' contributions

All authors were involved in writing/reviewing the manuscript. All authors approved the final manuscript.

## Consent

Written consent was obtained from the patient for presentation and publication of this manuscript.
